# Regulation of xylem cell fate

**DOI:** 10.3389/fpls.2014.00315

**Published:** 2014-07-01

**Authors:** Yuki Kondo, Takayuki Tamaki, Hiroo Fukuda

**Affiliations:** Laboratory of Cellular Biochemistry, Department of Biological Sciences, Graduate School of Science, The University of TokyoTokyo, Japan

**Keywords:** xylem, hormone, transcription factor, differentiation, patterning

## Abstract

The vascular system is organized throughout the plant body for transporting water, nutrients, and signaling molecules. During vascular development, xylem, phloem, and procambial/cambial cells are produced in a spatiotemporally organized manner. Several key regulators for xylem cell patterning and differentiation have been discovered, including auxin, cytokinin, CLE peptides, microRNAs, HD-ZIPIIIs, VNDs, and moving transcription factors SHR and AHLs. Recent studies are identifying functional interactions among these factors that ultimately determine xylem cell fate. This review focuses on regulatory networks underlying xylem cell fate determination in root vascular development.

## INTRODUCTION

Cell fate determination is a fundamental mechanism underlying complex morphogenesis in multicellular organisms. Vascular tissues consist of phloem, xylem, and procambial cells. In *Arabidopsis thaliana* roots, the pattern of the central xylem axis, two phloem poles, and their intervening procambium is maintained during development, suggesting a robust mechanism for determining the spatial fate of each vascular cell (Cano-Delgado et al., 2010). Therefore, root vascular development is considered as an excellent system for studying cell fate determination (Bonke et al., 2003; Kubo et al., 2005). Recent studies on root vascular development have uncovered novel machineries regulating xylem cell fates in roots, such as cell-to-cell communication mediated by ligand–receptor interaction and intercellular movement of transcription factors (Hirakawa et al., 2011; Miyashima et al., 2012). We summarize recent advances on xylem cell fate determination in roots and discuss the regulatory networks controlling xylem cell fate determination.

## CYTOKININ IS A CENTRAL REGULATOR OF PROTOXYLEM VESSEL CELL FATE IN ROOTS

**FIGURE 1:**
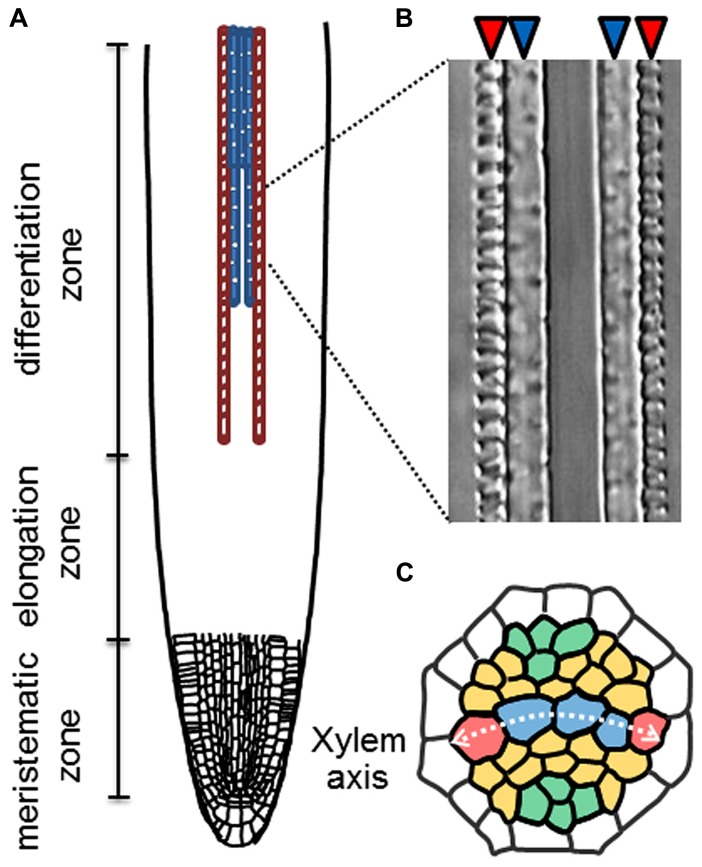
**Xylem cell specification in roots. (A)** Schematic illustration of longitudinal pattern of protoxylem (red) and metaxylem (blue) vessel cells in a root. **(B)** A Nomarski differential interference image of protoxylem (red arrowheads) and metaxylem vessels (blue arrowheads) in an *Arabidopsis* root. **(C)** Schematic illustration of radial cell patterning in the root stele. Red; protoxylem vessels (PX), blue; metaxylem vessels (MX), orange; procambial cells and green; phloem cells.

Root xylem vessels are classified into two types, protoxylem vessels and metaxylem vessels, which are equipped with a spiral-patterned and a pitted-patterned secondary cell-wall, respectively. The root vascular system is organized with precise cell patterning, in which five xylem vessel cells occupy the central xylem axis (**Figure 1**). In the xylem axis, two protoxylem vessels are always located on the outer side, and 2-4 metaxylem vessels are located on the inner side (**Figure 1**). It is widely recognized that root vascular cell identities are determined in the root apical meristem (RAM). The well-known vascular-specific marker genes *ALTERED PHLOEM DEVELOPMENT* (*APL*) and *ARABIDOPSIS HISTIDINE PHOSPHOTRANSFER PROTEIN 6* (*AHP6*) are expressed in phloem and protoxylem vessel cell files, respectively, in the RAM (Bonke et al., 2003; Mähönen et al., 2006).

**FIGURE 2:**
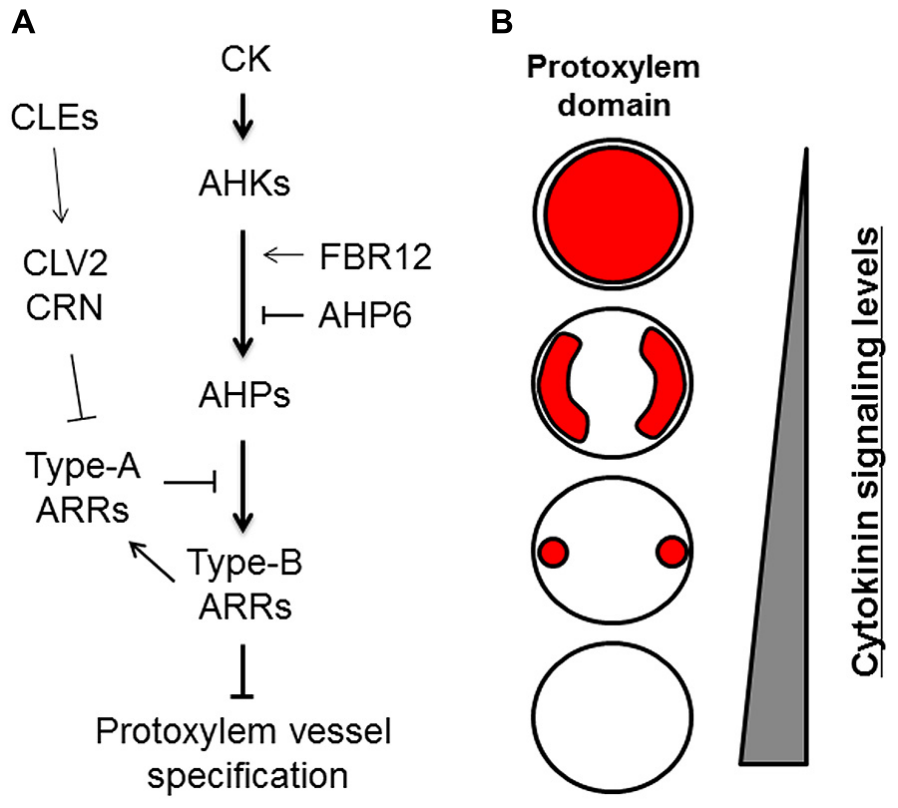
**Roles of cytokinin signaling in protoxylem vessel specification. (A)** Cytokinin (CK) signaling and its modulators involved in protoxylem vessel specification. **(B)** Schematic illustration of protoxylem domains (red) formed differentially depending on cytokinin signaling levels in the stele of roots.

The plant hormone cytokinin (CK) has been implicated in the specification of protoxylem vessels in roots (Mähönen et al., 2000; Fukuda, 2004). Application of the synthetic CK benzyladenine causes the loss of root protoxylem vessels in a dose-dependent manner (Yokoyama et al., 2007; Kondo et al., 2011; Ren et al., 2013). Conversely, reduction of CK content by expressing *CYTOKININ OXIDASE 2* (*CKX2*), which encodes a CK degradation enzyme, leads to the formation of extra protoxylem vessels (Mähönen et al., 2006). Thus, the number of protoxylem vessels depends on CK levels (**Figure 2**). CK signal transduction is initiated by the receptors *ARABIDOPSIS* HISTIDINE KINASE 2–4 (AHK2, AHK3, and CRE1/AHK4/WOL; Kieber and Schaller, 2014; **Figure 2A**). *AHK* mutants form extra protoxylem vessels adjacent to two original protoxylem vessels in the stele, due to the reduced CK sensitivity (Mähönen et al., 2006; Kondo et al., 2011; **Figure 2B**). The *wol* mutant displays a more severe phenotype, in which phloem cells are completely lost and only protoxylem vessels are formed in the stele (Scheres et al., 1995; Mähönen et al., 2006). The CK signal is transduced via phosphotransfer from AHKs to AHPs (**Figure 2A**). The *AHP* quintuple mutant (*ahp1 ahp2 ahp3 ahp4 ahp5*) exhibits the extra protoxylem phenotype similar to that of *ahk* mutants (Hutchison et al., 2006). These results indicate that CK negatively regulates protoxylem vessel formation via AHKs and AHPs. The atypical AHP, AHP6, lacks the histidine residue conserved among other AHPs. *AHP6* is expressed in future protoxylem vessel cell files in RAM, and loss-of-function mutants often cause a partial loss of protoxylem vessels (Mähönen et al., 2006). These results strongly suggest that AHP6 functions as a pseudo-phosphotransfer protein that interferes with phosphorelay from AHKs to AHPs by competing with other AHPs, leading to inactivation of CK signaling in protoxylem vessel formation (Mähönen et al., 2006; **Figure 2A**).

Typical AHPs activate transcription factors named type-B *ARABIDOPSIS* RESPONSE REGULATORs (ARRs), which are the final targets in CK signal transduction (Yokoyama et al., 2007; Argyros et al., 2008). Triple mutants of centrally acting type-B ARRs (*arr1 arr10 arr12*) develop ectopic protoxylem vessels similar to those of other CK-related mutants (Yokoyama et al., 2007; Ishida et al., 2008). Type-B ARRs directly up-regulate type-A *ARRs*, which negatively regulate CK signaling by interacting with AHPs and interfering with type-B ARR functions (To et al., 2007). Mutants for type-A *ARRs* exhibit elevated CK sensitivity (To et al., 2004) and have fewer protoxylem vessels in lateral roots but not in the primary root (Ren et al., 2009; Kondo et al., 2011). These studies indicate that the CK signaling cascade consisting of AHKs, AHPs, and ARRs has a central role in regulating protoxylem vessel cell specification (**Figure 2**).

## MODULATORS OF CYTOKININ SIGNALING REGULATE PROTOXYLEM VESSEL FORMATION

Modulators of CK signaling are involved in the regulation of protoxylem vessel formation. There are 32 genes encoding CLAVATA3/EMBRYO SURROUNDING REGION-related (CLE) peptides in *Arabidopsis* (Ito et al., 2006; Jun et al., 2008). Many CLE peptides, including CLE10, inhibit protoxylem vessel formation in wild-type plants (Kondo et al., 2011). By contrast, CLE10 does not inhibit protoxylem vessel formation in the type-B *arr10 arr12* double mutant (Kondo et al., 2011). Gene expression analysis shows that CLE10 down-regulates type-A *ARRs*. These results suggest that CLE10 activates CK signaling through the down-regulation of type-A *ARRs*, thereby suppressing protoxylem vessel formation (Kondo et al., 2011). Further genetic analysis suggests that CLAVATA2 (CLV2) may act as a receptor that mediates CLE10 signaling and regulates protoxylem vessel formation (Kondo et al., 2011).

A recent study reported that the loss-of-function mutant of *FUMONISIN B1-RESISTANT 12* (*FBR12*) produced extra protoxylem vessels due to reduced CK sensitivity (Ren et al., 2013). *FBR12* encodes a eukaryotic translation initiation factor (elF5A) that is believed to play various roles via interactions with different proteins and RNAs (Thompson et al., 2003; Jao and Chen, 2006; Feng et al., 2007). FBR12 physically and genetically interacts with CRE1/AHK4/WOL and AHPs, which results in enhanced CK signaling (Ren et al., 2013). The modulation of CK signaling by various factors at different signaling steps enables fine spatiotemporal regulation of the protoxylem vessel domain (**Figure 2**).

## SPATIAL REGULATION OF THE CYTOKININ ACCUMULATION DOMAIN IN ROOTS

**FIGURE 3:**
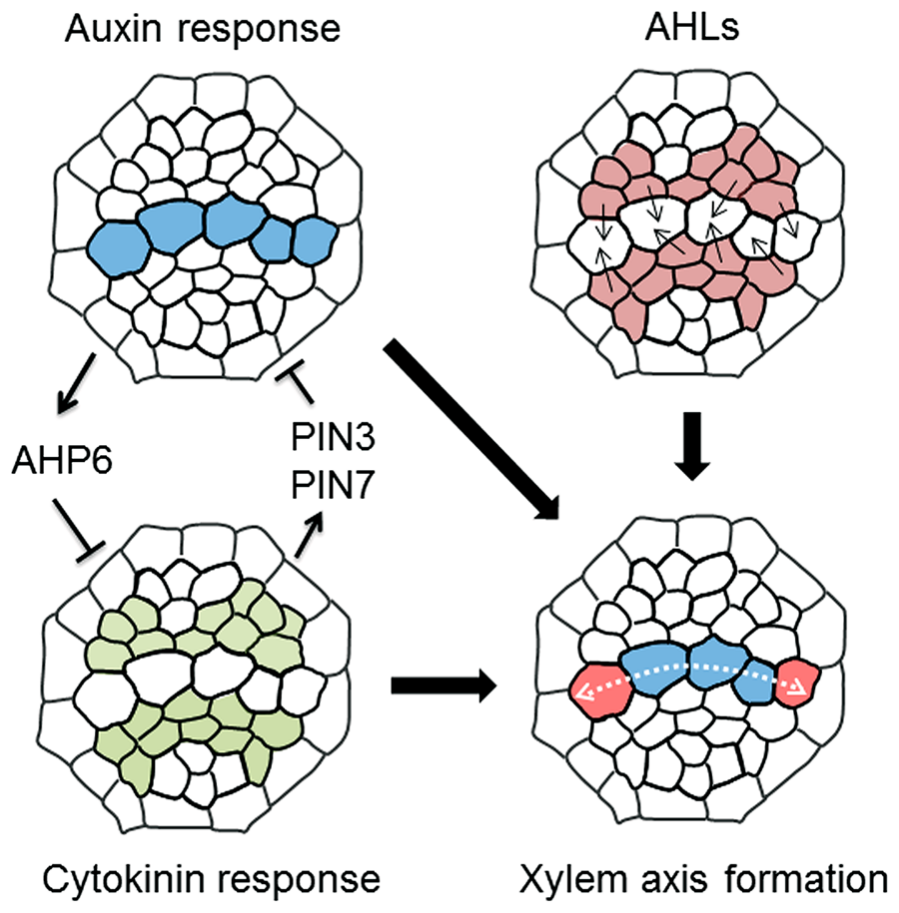
**A molecular mechanism determining xylem axis domain.** Schematic illustration showing a machanism underlying the formation of xylem axis domain in the root stele. (1) Mutual inhibition of high auxin and cytokinin domain defines protoxylem vessel domain, in which auxin inhibits cytokinin signaling via AHP6 and cytokinin restricts the auxin accumulation via PIN3 and PIN7. (2) AHLs movement plays a role in the determination of not only protoxylem vessel but also metaxylem vessel domain, thereby establishing the boundary between xylem axis and procambium domain in root stele. (3) The combination of these regulations leads to the formation of spatially regulated xylem axis domain. The details are described in the main text.

Precise protoxylem vessel patterning requires spatial control of CK accumulation. Reporter-GUS analysis using hormone-response markers shows that domains with high auxin and high CK levels are localized in xylem axis and procambium in the stele, respectively (Bishopp et al., 2011a; **Figure 3**). The auxin distribution pattern is formed by auxin lateral transport through auxin efflux carriers PIN-FORMED 3 (PIN3) and PIN7 (Bishopp et al., 2011a). High auxin level in the xylem axis directly up-regulates *AHP6* expression in the protoxylem vessel position via auxin-responsive elements in its promoter (Bishopp et al., 2011a). NPA treatment, which inhibits polar auxin transport, blocks *AHP6* expression in the protoxylem vessel position, resulting in the loss of protoxylem vessels (Bishopp et al., 2011a). AHP6 has a negative role in CK signaling (Mähönen et al., 2006). These results indicate that PIN-mediated polar auxin transport and auxin accumulation induces *AHP6* expression, which in turn attenuates CK signaling at the protoxylem vessel position (**Figure 3**).

Conversely, *PIN7* expression is regulated by CK (Bishopp et al., 2011a). The *PIN7* expression domain overlaps the high CK-response domain, and *PIN7* transcript levels are increased in response to CK treatment (Bishopp et al., 2011a). A recent study discusses a new technique for blocking symplastic connections by inducing the expression of mutated *CALLOSE SYNTHASE 3* (*CALS3*), which substantially increases callose deposition at plasmodesmata (Vaten et al., 2011). Using this technique to inhibit symplastic transport revealed that basipetal transport of CK via the phloem is required for controlling the *PIN7* expression domain (Bishopp et al., 2011b; Vaten et al., 2011). This result indicates that basipetal CK transport toward the RAM restricts the high auxin-response domain in the xylem axis by modulating auxin lateral transport (Bishopp et al., 2011b). Consequently, the fact that the* ahk3 cre1* double mutant forms ectopic protoxylem vessels adjacent to the original protoxylem vessels (Mähönen et al., 2006; Kondo et al., 2011) can be explained because the high auxin-response domain in the xylem axis expands due to reduced CK signaling in that mutant (Mähönen et al., 2006; Bishopp et al., 2011a, b; Kondo et al., 2011; **Figure 2B**). Therefore, this mutually inhibitory feedback loop between auxin and CK allows precise establishment and maintenance of the protoxylem vessel position (**Figure 3**).

## FACTORS THAT REGULATE METAXYLEM VESSEL FORMATION

The protoxylem domain is determined by the balance between auxin and CK; however, the molecular mechanisms that determine the metaxylem vessel domain remain unclear. Recently, Ursache et al. (2014) isolated mutants defective in *TRP2*, which is involved in tryptophan biosynthesis and tryptophan-mediated auxin biosynthesis. These mutants have a defect in metaxylem vessel formation but not in protoxylem vessel formation, suggesting an involvement of auxin biosynthesis in metaxylem vessel formation.

The conserved CLE–WOX signaling pathway is involved in metaxylem vessel development in rice (*Oryza sativa*; Chu et al., 2013). A rice CLE peptide named FON2-LIKE CLE PROTEIN2 (FCP2) negatively controls the expression of *quiescent-center-specific-homeobox* (*QHB*), which is an ortholog of AtWOX5 and is expressed in the QC and metaxylem precursor cells (Kamiya et al., 2003; Chu et al., 2013). Negative regulation of QHB by application of exogenous FCP2 causes the loss of metaxylem identity, leading to aberrant cell division in the metaxylem vessel position (Chu et al., 2013). As mentioned previously, CLE peptides can inhibit protoxylem vessel formation in *Arabidopsis* (Kondo et al., 2011). Therefore, the role of CLE signaling in the regulation of root xylem development is not conserved between *Arabidopsis* and rice.

## BOUNDARY FORMATION BETWEEN XYLEM AND PROCAMBIUM DOMAINS

A recent study reported that the boundary between the procambium and xylem axis is determined by moving transcription factors named AT-HOOK MOTIF NUCLEAR LOCALIZED PROTEINs (AHLs; Zhou et al., 2013; **Figure 3**). In *ahl3* and *ahl4* single mutants, both ectopic protoxylem vessels and ectopic metaxylem vessels are formed in the procambial region adjacent to the xylem axis, suggesting an enlargement of the xylem axis domain (Zhou et al., 2013). This phenotype has some similarity with that of CK-defective mutants in terms of excess protoxylem vessel formation, but is distinctive in terms of extra metaxylem vessel formation adjacent to the original metaxylem. *AHL4* is expressed in the procambium and its protein product can move into the xylem axis domain (Zhou et al., 2013). This intercellular movement is required for correct boundary formation between the procambium and the xylem (Zhou et al., 2013). The* ahl3 ahl4* double mutant does not exhibit a more severe xylem phenotype compared with those of the single mutants, indicating that AHL3 and AHL4 function together (Zhou et al., 2013). AHL3 and AHL4 form a heterodimer, and have the potential to move from the procambium to the xylem (Zhou et al., 2013). High auxin and high CK-response domains are altered in *ahl* mutants (Zhou et al., 2013), but the relationship between AHLs and hormonal regulation of xylem axis formation is unknown. Further analysis of the function of AHLs may provide new insights into the mechanisms underlying boundary formation between xylem axis and the procambium.

## MOLECULAR SWITCHES FOR PROTOXYLEM AND METAXYLEM VESSEL CELL FATE

**FIGURE 4:**
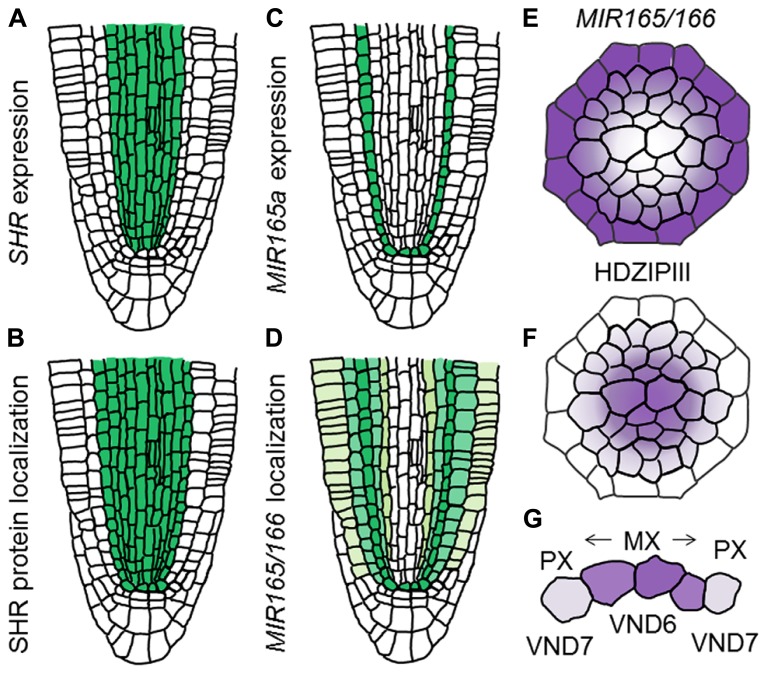
**Determination of xylem vessel types in the xylem axis. (A–D)** Schematic illustration showing difference between gene expression and localization of gene products for *SHR* and *MIR165/166* in the RAM (based on the data reported in Carlsbecker et al., 2010). **(A)** Expression pattern of *SHR*. **(B)** Localization of SHR protein. **(C)** Expression pattern of *MIR165a*. **(D)** Localization of *MIR165/166*. **(E,F)** Schematic illustration showing localization patterns of *MIR165/166* **(E)** and HD-ZIPIII transcripts **(F)**. **(G)** Schematic illustration of xylem vessel type determination. Vessel types are determined by VND6 and VND7 as well as HD-ZIPIII gradients.

The GRAS-family transcription factor SHORT-ROOT (SHR), which is known to establish the identity of endodermis and cortex (Helariutta et al., 2000; Nakajima et al., 2001; Gallagher et al., 2004; Cui et al., 2007), also functions in the regulation of protoxylem and metaxylem specification (Carlsbecker et al., 2010). The *shr* mutant forms metaxylem vessels at the protoxylem position, indicating a switch of vessel types from protoxylem to metaxylem (Carlsbecker et al., 2010). SHR moves from the stele to the endodermis and induces expression of *miR165* and *miR166*, in co-operation with SCARECROW (SCR; Carlsbecker et al., 2010; **Figures 4A–C**). Then, *miR165* and *miR166* move from the endodermis to the stele, leading to their higher accumulation in the outer region than in the inner domain of the stele (Carlsbecker et al., 2010; **Figures 4D,E**). *miR165* and *miR166* destabilize the mRNAs of *class III homeodomain-leucine zipper* (*HD-ZIPIII*) family genes, which include *AtHB8*, *PHABULOSA* (*PHB*), *PHAVOLUTA*, *REVOLUTA*, and *CORONA/AtHB15* (Prigge et al., 2005; Carlsbecker et al., 2010). This results in higher expression of these genes in the inner domain of the stele (Carlsbecker et al., 2010; **Figure 4F**). The *miR165/miR166*-insensitive mutant *phb-7d* exhibits ectopic metaxylem vessel formation at the protoxylem vessel position, similarly to that of the *shr* mutant (Carlsbecker et al., 2010). Conversely, quadruple mutants for HD-ZIPIIIs only produce protoxylem vessels in the xylem axis, which is confirmed by the loss of metaxylem vessel marker ACAULIS 5 (ACL5) and ectopic expression of protoxylem vessel marker AHP6 (Mähönen et al., 2006; Muniz et al., 2008; Carlsbecker et al., 2010). These results indicate that *HD-ZIPIII* genes ultimately determine the xylem vessel types; high expression induces metaxylem vessels, whereas low expression induces protoxylem vessels (Miyashima et al., 2011).

VASCULAR-RELATED NAC-DOMAIN 6 (VND6) and VND7, which belong to the NAM, ATAF1/2, and CUC2 (NAC) transcription factor family, are master regulators for xylem cell differentiation and determine the cell fate of the metaxylem and protoxylem vessels, respectively (Kubo et al., 2005). Both VND6 and VND7 directly up-regulate genes involved in programmed cell death and secondary cell-wall thickening (Ohashi-Ito et al., 2010; Yamaguchi et al., 2011). *VND6* is expressed in the central metaxylem vessels, whereas *VND7* is expressed primarily in the protoxylem vessel cell files (Kubo et al., 2005). Overexpression of *VND6* and *VND7* leads to ectopic formation of metaxylem and protoxylem vessel elements, respectively (Kubo et al., 2005; Yamaguchi et al., 2010a). Conversely, expression of *VND6* and *VND7* fused with the chimera repression domain SRDX under control of their own promoters causes a failure of central metaxylem and protoxylem vessel development, respectively (Kubo et al., 2005). However, loss-of-function mutants for *VND6* and* VND7* do not show any defect in root xylem development (Kubo et al., 2005), indicating that seven VND family members function differently but in some cases redundantly in the regulation of xylem cell differentiation. VND-interacting 2 (VNI2) was identified as an interacting protein with VND7 by a yeast two-hybrid screen (Yamaguchi et al., 2010b). VNI2 negatively regulates xylem vessel differentiation in opposition to VND7 (Yamaguchi et al., 2010b). Further analyses are required to elaborate the relationship between VNDs, VNIs, and HD-ZIPIIIs in the context of switching xylem vessel types (**Figure 4G**).

## CONCLUDING REMARKS

Xylem cell fate is regulated by spatiotemporal actions of various signaling factors. Mutual inhibition between CK and auxin determines the precise xylem vessel domains, in particular protoxylem vessels. Some CLE peptides play a role in fine-tuning the CK signal. The movement of AHLs defines the boundary between the procambial domain and the xylem domain, thereby establishing the xylem axis in root stele. The opposite movement of SHR and *miR165/miR166* between outer endodermis and inner stele ultimately regulates the level of HD-ZIPIII proteins, resulting in the fate determination of different xylem vessel types. Finally, the master transcription factors VND6 and/or VND7 execute the program of metaxylem and protoxylem vessel differentiation, respectively. Collectively, in roots, xylem cell fates are controlled precisely by a regulatory network consisting of hormone signaling pathways and transcription factors in a hierarchical organization.

However, the basic root vascular pattern is determined during embryogenesis. Therefore, to understand the regulation of xylem cell fate, we should elucidate the mechanism underlying the onset of vascular cells in early embryos. Recent studies demonstrated that two bHLH transcription factors, LONESOME HIGHWAY (LHW) and TARGET OF MONOPTEROS 5 (TMO5), play crucial roles in the initiation of vascular cells (De Rybel et al., 2013; Ohashi-Ito et al., 2013). Further functional analysis of downstream targets of these transcription factors may provide novel insights into understanding the determination of xylem cell fates.

## Conflict of Interest Statement

The authors declare that the research was conducted in the absence of any commercial or financial relationships that could be construed as a potential conflict of interest.
